# In situ delivery of biobutyrate by probiotic *Escherichia coli* for cancer therapy

**DOI:** 10.1038/s41598-021-97457-3

**Published:** 2021-09-13

**Authors:** Chung-Jen Chiang, Yan-Hong Hong

**Affiliations:** 1 Department of Medical Laboratory Science and Biotechnology, China Medical University, No. 91, Hsueh-Shih Road, Taichung, Taiwan 40402; 2 Department of Chemical Engineering, Feng Chia University, Taichung, Taiwan 40724

**Keywords:** Cancer therapy, Biotechnology, Cancer, Cell biology, Oncology

## Abstract

Butyrate has a bioactive function to reduce carcinogenesis. To achieve targeted cancer therapy, this study developed bacterial cancer therapy (BCT) with butyrate as a payload. By metabolic engineering, *Escherichia coli* Nissle 1917 (EcN) was reprogrammed to synthesize butyrate (referred to as biobutyrate) and designated EcN-BUT. The adopted strategy includes construction of a synthetic pathway for biobutyrate and the rational design of central metabolism to increase the production of biobutyrate at the expense of acetate. With glucose, EcN-BUT produced primarily biobutyrate under the hypoxic condition. Furthermore, human colorectal cancer cell was administrated with the produced biobutyrate. It caused the cell cycle arrest at the G1 phase and induced the mitochondrial apoptosis pathway independent of p53. In the tumor-bearing mice, the injected EcN-BUT exhibited tumor-specific colonization and significantly reduced the tumor volume by 70%. Overall, this study opens a new avenue for BCT based on biobutyrate.

## Introduction

Cancer is a life-threatening disease. The medical treatment of this disease conventionally relies on chemotherapy and radiotherapy. However, these conventional therapies usually fail to prevent tumor recurrence. Tumor vasculature consists of irregular and poorly-organized blood vessels which inadequately supply nutrients and oxygen. This in turn leads to the development of hypoxic or anoxic regions in malignant tumors. Hypoxic cells are recalcitrant to the treatment of radiotherapy^1^. In addition, chemotherapeutic agents often show a poor treatment efficacy because they are unable to target and penetrate deep into tumor tissues^2,3^. This problem can be in part solved by the technology of targeted therapy that implements a nanoscale carrier for specific delivery of therapeutic cargos to tumor sites^4^. Nevertheless, the issue of drug diffusibility still needs to be addressed^5,6^.

Tumor-targeting bacteria including *Bifidobacterium*^7^, *Clostridium*^8^, *Salmonella*^9^, and *Escherichia*^10^ have been implemented for cancer therapy, known as bacterial cancer therapy (BCT). The mechanism through which various bacteria colonize tumors is diverse. For instance, the initial inflammation response mediated by TNF facilitates the accumulation of *Salmonella* in tumors^11^. In a more general way, bacteria employ chemotaxis to sensitize the specific receptors and are driven to the tumor microenvironment^12,13^. They proliferate in the hypoxic/necrotic regions of tumor tissues and evade clearance by the immune system^14^. The concept of BCT stems from William B. Coley who illustrated the induction of tumor regression by “Coley’s toxin”^15^. The advance in synthetic biology further revolutionizes the technology of BCT, which generally enables genetically-modified bacteria to synthesize and deliver an array of bioactive payloads in situ^16^.

BCT has been implemented with tumor-seeking bacteria that synthesize a variety of payloads, mainly including prodrugs-converted enzymes^17^, short hairpin RNA^18^, cytokines^19^ antigens^20^, antibodies^21^, and bacterial toxins^22^. Most of these payloads have the intrinsic limitation of poor diffusibility or/and proliferating cell-specificity. Bacterial toxin has an advantage over others that it is secreted from the bacterial vector after being synthesized, directly lyses cancer cells, and passively diffuses into tumor tissues. As illustrated by previous studies, the administration of hemolysin E-producing *S. typhimurium* and *Staphylococcus aureus* α-hemolysin-expressing *E. coli* significantly decreased the volume of 4T1 tumor^23^ and MCF7 tumor^24^, respectively. However, it needs to perform the bacterial delivery of toxins with caution because an inadequate regulation of toxin expression is detrimental to healthy cells. Tumor specificity appears to be a desired trait for a promising payload. Short-chain fatty acids (SCFAs) are of particular interest, and they are produced in intestinal microbiota by fermentation of dietary fibers^25^. Among SCFAs, butyrate plays the most important role in intestinal physiology. It provides more than 70% of the energy to fuel the metabolic activity of coloncytes while lowers colorectal oncogenesis by modulation of signaling pathways to induce apoptosis of cancer cells^26,27^. However, the clinical implementation of butyrate is restricted by its rapid clearance and low bioavailability in the circulation. This issue has been addressed by exploration of butyrate analogues as chemotherapeutic agents^28^ and of lipid nanoparticles (NPs) for delivery of butyrate^29^. The in vitro study showed that the administration of butyrate-loaded NPs increased the anti-cancer efficacy in a cell type-dependent way^30^. Another study developed the cholesteryl butyrate (cholbut) NPs-based delivery system, which consequently inhibited adhesion and migration of colon cancer cells^31^. In the mice model, the tumor growth was significantly retarded by cholbut NPs^32^. Nevertheless, the practical application of cholbut NPs appears to be limited by tumor specificity.

A robust carrier that selectively delivers butyrate manifests its potential application for targeted cancer therapy. BCT apparently serves as a promising candidate. Following the administration, engineered bacteria selectively colonize in tumors and constantly produce the therapeutic agent on site. Without the need of prior purification, the therapeutic payload is directly delivered to cancerous sites to improve the treatment efficacy. *E. coli* Nissle 1917 (EcN) is a probiotic strain without producing any enterotoxins and cytotoxins. This bacterium has been applied as a living therapeutic for the treatment of various gastrointestinal illnesses involving diarrhea and colitis ulcerosa^33^. As illustrated with 4T1 breast tumor, EcN exhibited tumor-specific colonization and replication in an efficient manner. In particular, the efficiency of tumor colonization remained unaffected with either the intraperitoneal or intratumoral injection of EcN^34^. Taking advantage of the EcN’s characteristic nature, this study aimed to develop a BCT platform for in situ delivery of butyrate. By metabolic engineering, EcN was equipped with a heterogenous pathway responsible for the synthesis of butyrate (hereinafter referred to as biobutyrate). Consequently, the administration of the biobutyrate-producing EcN effectively induced tumor regression in mice xenografted with the human colorectal cancer cell. To our best knowledge, this study first reports the potential application of BCT based on biobutyrate.

## Materials and methods

### Genetic modification of bacterial vectors

Probiotic EcN was genetically modified as follows. Firstly, the genome of EcN was integrated with a gene cluster containing *phaA* of *Cupriavidus necator* and *hbd* of *Clostridium acetobutylicum* DSM792 under the control of λP_L_ promoter (PλP_L_) (PλP_L_-*phaA*-*hbd*). This was conducted with the suicide plasmid pPhi-PhaAHbd^35^, which enables insertion of the DNA carrying PλP_L_-*phaA*-*hbd* into ϕ80 *attB* by ϕ80 integrase under the nonpermissive condition^36^. The kanamycin-resistance determinant flanked by the *loxP* site was removed by the Cre recombinase using the helper plasmid pTH19-CreCs^37^. Secondly, the genomic insertion of the PλP_L_-driven *crt* of *C. acetobutylicum* was carried out with the conditional plasmid pLam-Crt^35^. Thirdly, an extra ϕ80 *attB* site was generated with plasmid pBlue-P80Gn according to the reported protocol^37^. The PλP_L_-regulated *ter* of *Terponema denticola* DSM14222 was integrated at ϕ80 *attB* site by using the conditional plasmid plasmid pPhi-Ter^35^. Finally, the endogenous *atoD* was fused with PλP_L_ to enhance its expression. To do so, the DNA containing PλP_L_ with two homologous regions was amplified from plasmid pSPL-atoD by PCR with RC13034-RC13035 primers^35^. Following electroporation, the PCR DNA was integrated into the target site through the λ Red-mediated homologous recombination. The genetic construction resulted in a bacterial strain, designated EcN-ato.

EcN-ato was further modified in the following. To enhance the expression level, *aceEF* was fused with PλP_L_ (PλP_L_-*aceEF*) by using plasmid pPR-aceE and RC12060-RC12086 primers as previously reported^38^. In addition, endogenous *adhE* and *pta* were inactivated. This was carried out by amplification of the DNA containing △*adhE::FRT-kan-FRT* or △*pta::FRT-kan-FRT* from JW1228-1 (△*adhE748::kan*) and JW2294-1 (△*pta779::kan*)^39^ with primers Adh1-Adh2 (tcttgcttacgccacctgg and ctgcaaatagttgtgcagagg) and Pta1-Pta2 (gcacgtttcggcaaatctgg and gtggtaagtatgcaaagtgg), respectively. With the aid of λ Red, the PCR DNA was individually integrated to knock out the target gene after electroporation. The removal of *kan* was conducted with the Flp recombinase with helper plasmid pCP20^40^. Consequently, the construction produced a strain designated EcN-BUT.

### Bacterial fermentation

To investigate the biobutyrate production, bacteria were cultured under the hypoxic condition (~ 1%–2% oxygen) essentially following our previous report^35^. In brief, bacteria were grown in a sealed flask containing TB medium (12 g/L tryptone, 24 g/L yeast extract, 2.13 g/L KH_2_PO_4_, and 12.54 g/L K_2_HPO_4_) supplemented with 5 g/L glucose. The bacterial culture was maintained at 37 °C and the fermentation proceeded for 24 h. At the end of the experiment, the products in the culture broth were processed for the analysis by using high-performance liquid chromatography (HPLC) AS3000 (Thermo Fisher Scientific, MA, USA) which was equipped with an autosampler as described previously^35^.

### Cell culture

HT29 cell (ATCC HTB-38) was cultured in Dulbecco’s modified Eagle's medium supplemented with 10% fetal calf serum (Thermo Fisher Scientific) and 1% penicillin/streptomycin. The cell culture was maintained at 37 °C in an atmosphere supplemented with 5% CO_2_.

### Cell viability assay

Cell proliferation was determined by the 3-(4,5-dimethylthiazol-2-yl)-2,5-diphenyltetrazolium bromide (MTT) (Invitrogen, CA, USA) assay according to the previous report^41^. Tumor cells were seeded in 96-well microplates at 1 × 10^4^ cells per well for 12 h and then treated with various concentrations of biobutyrate. After the treatment, tumor cells in the plate were washed twice with 10 mM phosphate buffered saline (PBS) at pH 7.4. MTT (5 mg/mL) in PBS was added to each plate for 4 h. Following the removal of culture supernatant, the same volume of 10 mM dimethylsulfoxide (DMSO) was replenished in each plate which was incubated on a microplate shaker for 10 min. The absorbance at 570 nm was measured using BioRad 680 microplate reader (Hercules, CA, USA).

### Real-time digital bio-imaging

Tumor cells were seeded into a 96-well plate (2 × 10^4^ cells per well) and incubated with various concentrations of either butyrate or biobutyrate upon reaching 40% confluence. The cell imaging was carried out by placing the plate in the multi-mode plate reader Cytation 5 (Biotek, Winooski, VT). The assay was performed at 37 °C in an atmosphere supplemented with 5% CO_2_. Images of each well were collected every 24 h over 3 day by using a 10X phase-contrast objective. Cell images were processed with GEN5 software (Biotek).

### DNA fragmentation analysis with DAPI fluorescence staining

Tumor cells were seeded in 24-well microplates at 1 × 10^4^ cells per well for 12 h and then treated with various doses of biobutyrate. After washing with PBS, tumor cells were fixed with ice-cold 4% paraformaldehyde in PBS for 20 min and then stained with 0.2 μg/mL 4,6-diamidino-2-phenylindole (DAPI) (Sigma-Aldrich, MO, USA) for 10 min. Tumor cells were washed with PBS and were sealed in VECTASHIELD Antifade Mounting Medium (Vector Lab., CA, USA). Nuclear morphology was observed by using fluorescent microscope (Olympus, Nagano, Japan).

### Acridine orange/ethidium bromide (AO/EB) staining

The AO/EB staining was performed following the reported protocol^42^. In brief, the supernatant (containing culture medium and floating HT29 cells) was transferred to a small tube. The rest of adherent cells were detached with PBS plus 1 mM ethylenediaminetetraacetic acid (EDTA). Both cell samples were pooled together in the tube to which the AO/EB dye mixture (100 μg/mL each in PBS) was added. The analysis was then conducted with an inverted microscope (Nikon eclipse TS100) at 400 × magnification with excitation filter 480/30 nm, dichromatic mirror cut-on 505 nm LP, and barrier filter 535/40 nm. Photographs were taken with a digital camera (Nikon COLPIX). More than 100 cells in each preparation were counted, and the examination was carried out in triplicate.

### Cell cycle analysis

The analysis method essentially followed the reported protocol^43^. Tumor cells were seeded in 6-well microplates at 1.2 × 10^5^ cells per well for 12 h, followed by treatment with various biobutyrate for 72 h. Cells were harvested with trypsin (Invitrogen), washed with PBS twice, and fixed with ice-cold ethanol (70%) for 2 h at − 20 °C. Fixed cells were washed twice in cold PBS and resuspended in 300 μL of freshly prepared PBS plus 0.1% Triton X-100, 0.2 mg/mL DNase-free RNase A (Sigma-Aldrich), and 10 μg/mL propidium iodide (PI) (Roche, IN, USA). After incubation at 37 °C in the dark for 20 min, cells were filtered through nylon mesh (BD Biosciences, CA, USA). PI-stained cells were loaded and analyzed by the FACScanto flow cytometer system (BD Biosciences) with the excitation at 536 nm and emission at 617 nm. Data were analyzed by using the FCS Express 4 Flow Software (De Novo Software).

### Apoptosis analysis

Tumor cells were seeded in 6-well microplates at 1.2 × 10^5^ cells per well for 12 h and then treatmented with various biobutyrate. The treated cells were examined with Annexin V-FITC Apoptosis detection kit (Sigma-Aldrich) following the manufacturer’s instruction. Annexin V-FITC and PI were added sequentially to cells which were resuspended in the binding buffer. Cells were incubated for 15 min in the dark at room temperature. The analysis was then conducted with the FACSCalibur flow cytometer (BD Biosciences). Data were analyzed by using the Cell Quest software (BD Biosciences).

### Immunoblotting analysis

Tumor cells that received the treatment in 6-well plates were scraped and placed in RIPA buffer (150 mM NaCl, 1.0% IGEPAL CA-630, 0.5% sodium deoxycholate, 0.1% SDS, and 50 mM Tris, 1 mM PMSF) (Sigma-Aldrich). Protein concentrations were determined by BCA Protein Assay Reagent kit (Sigma-Aldrich). By denaturing at 95 °C for 10 min, protein samples (40 μg) were resolved on the SDS-PAGE gel and then transferred to PVDF membranes (Millipore, MA, USA). Membranes were blocked in 5% fat-free milk in TBST buffer (Tris-buffered saline with 0.1% Tween 20 detergent) for 2 h and incubated with primary antibodies, including p53 (sc-126); Bcl-2 (sc-7382); GAPDH (sc-477724); Cytochrome C (sc-13156); PARP (sc-74470); cyclin E (sc247), caspase-3 (sc-7272) (Santa Cruz Biotech., CA, USA); p21 (2947 s); CDK4 (12790S); caspase 9 (9508); cleaved caspase 3 (9661); Bax (2772 s) (Cell signaling Tech., MA, USA). The reaction proceeded overnight at 4 °C. After rinsing with TBST buffer, membranes were incubated with HRP-conjugated secondary antibodies (Jackson ImmunoResearch Lab., PA, USA) for 1 h. Immunoblotting results were analyzed by enhanced chemiluminescence detection system based on ECL-Plus kit (Amersham, Germany). Protein levels were quantified with the luminescent image analyzer LAS 3000 (Fuji Film, Tokyo, Japan) and normalized to the GAPDH level.

### Murine tumor model

Animal experiments exclusively complied with the Guide stipulated by the Council of Agriculture Executive Yuan and ARRIVE guidelines for the Care and Use of Laboratory Animals and were approved by the Animal Ethics Committee of China Medical University (No. 2016-029). Specific-pathogen-free (SPF) BALB/cAnN.Cg nude male mice (4 weeks old and the body weight of 20 g) were obtained from the National Laboratory Animal Center, Taiwan. The xenograft mouse models were established by implanted with the subcutaneous injection of 1 × 10^7^/100 µL HT29 cells (human colorectal cancer, ATCC HTB-38) on the right flank side. The development of tumor was monitored along the time course and the tumor volume was calculated based on the caliper measurement. Bacteria were grown aerobically on TB medium at 37 °C for 10 h. At the end of the culturing, bacteria were harvested by centrifugation and washed with PBS. Following centrifugation, bacteria pellets were resuspended in PBS for further use. EcN (10^6^/100 µL), or EcN-BUT (10^6^/100 µL) was injected into the peritumoral region once the tumor volume reached approximately 100 mm^3^. The injection was performed twice a week. At the end of experiments, mice were sacrificed to have their tumors and organs analyzed.

To analyze the distribution of bacteria, mice organs were weighed and homogenized aseptically in ice-cold PBS. The homogenate was serially diluted and plated onto LB agar plates. After incubation at 37 ℃ for 24 h, the colony forming unit (CFU) per gram tissue was determined by dividing bacterial by the weight of the organ.

### Histopathological study

The histopathological study was conducted with the hematoxylin and eosin (H&E) stain^41^. Mice were subjected to euthanasia by transcardial perfusion with 4% paraformaldehyde in PBS and stored in the 70% ethanol solution. Tumors were blocked in longitudinal sections and processed for paraffin embedding, followed by cutting with a microtome (Leica Microsystems Inc., IL, USA). H&E stains were applied to tumor sections with the thickness of 5 μm, and the images were photographed using a light microscopy (Olympus).

The immunohistochemical (IHC) analysis of cleaved caspase-3 was carried out following the previous report^44^. In brief, the retrieval of antigens was performed using Proteinase K (Dako, CA, USA). Samples were permeabilized with 0.05% Triton-X in PBS for 5 min. Protein Block Solution (Abcam, MA, USA) was applied for non-specific antigen blocking. Sample slides were then incubated with the primary antibody against cleaved caspase-3 (Cell Signaling Tech.), and the result was analyzed by using the EnVision kit (Dako). Slides were mounted in MM24 (Leica, Germany). Digital images of whole-tissue sections were acquired using a Cytation 5 (Biotek, VT, USA). Ten images from each slide were selected at random at 40 × magnification using GEN5 software (Biotek).

### Statistical analysis

Statistical analyses were performed with PRISM7 (GraphPad, La Jolla, CA) and graphs were prepared using SigmaPlot 10.0 (Systat Software, San Jose, CA). Values were expressed as mean ± standard deviation (SD) taken from three independent experiments. Results among groups were analyzed by one-way analysis of variance (ANOVA) based on Holm-Sidak test to investigate the significant difference between the treatment and the control group. The Tukey’s multiple comparison test was applied to all studies. The statistical significance was acknowledged at the level of ∗p < 0.05, ∗∗p < 0.01, and ∗∗∗p < 0.001.

## Results

### Design of the bacterial vector

**Figure 1:**
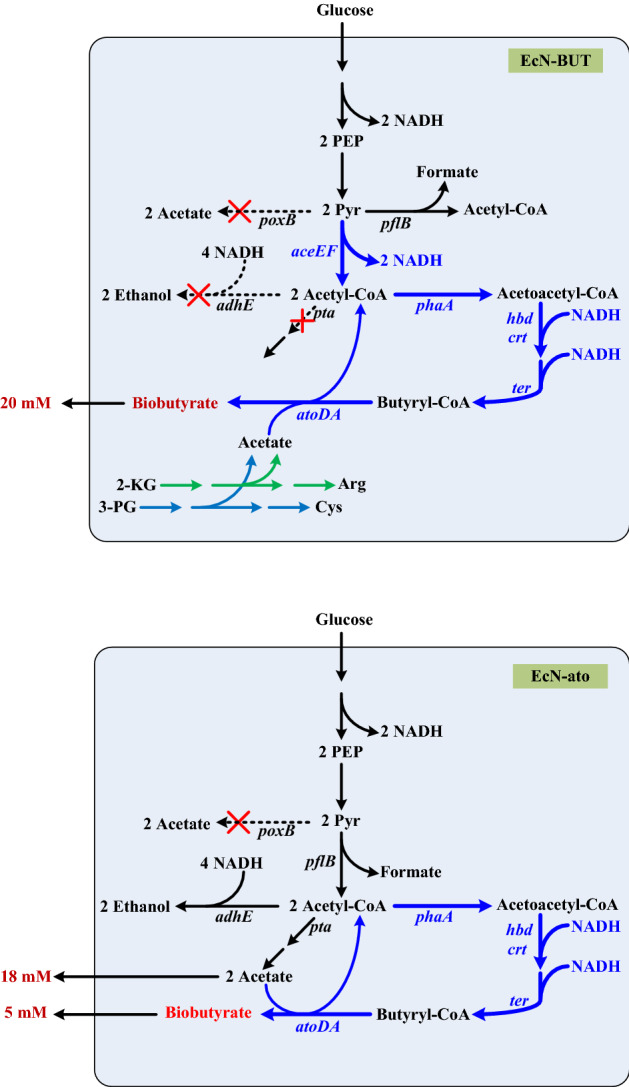
Schematic illustration of the metabolic pathway leading to biobutyrate from glucose. The strategy of metabolic engineering was applied to rewire EcN metabolism for the synthesis of biobutyrate. Pathways of enhancement and of new construction were indicated by solid lines (blue) while pathways of blockage were marked with “X” (dotted lines). The genetic construction resulted in the bacterial vector including EcN-BUT (top) and EcN-ato (bottom). After the fermentation, the bacterial production of acetate (mM) or biobutyrate (mM) was indicted in the Figure. Acetate is produced during the biosynthesis of Arg from 2-KG and of Cys from 3-PG in EcN-BUT. The genes involves in the metabolic pathway: *aceEF* pyruvate dehydrogenase complex, *adhE* aldehyde-alcohol dehydrogenase, *atoDA* acetoacetyl-CoA transferase, *pta* phosphate acetyltransferase, *poxB* pyruvate oxidase, *phaA* β-ketothiolase, *hbd* 3-hydroxybutyryl-CoA dehydrogenase, *crt* crotonase, *ter* trans-enoyl-CoA reductase. *Arg* argine, *Cys* cysteine, *2-KG* 2-ketoglutarate, *PEP* phosphoenolpyruvate, *3-PG* 3-phosphoglycerate, *Pry* pyruvate.

The synthetic pathway of biobutyrate was constructed in EcN as follows (Fig. 1). The heterologous gene cluster including *phaA*, *hbd*, *crt*, and *ter* under the control of PλP_L_ was integrated into the bacterial genome with the aid of the phage-integration system. This artificial pathway leads to butyryl-CoA from glucose. Next, endogenous *atoDA* was overexpressed by fusion with PλP_L_. The function of *atoDA* converts butyryl-CoA to biobutyrate at the expense of acetate. The genetic construction resulted in a bacterial vector, designated EcN-ato. The production of biobutyrate was investigated in EcN-ato grown on glucose. The analysis showed that the bacterial strain produced 5 mM biobutyrate and 18 mM acetate at 24 h.

The balanced redox state in the bacterial strain favors the fermentative production of biobutyrate^35^. During fermentation, *pflB* functions to provide acetyl-CoA. Pyruvate dehydrogenase (PDH) directs pyruvate into acetyl-CoA associated with the production of NADH (Fig. 1). Therefore, the expression of endogenous *aceEF* was driven by PλP_L_ to increase the PDH activity in EcN-ato. The reaction step competing for acetyl-CoA was further blocked by deletion of *adhE*. These strategies aim to increase the intracellular level of NADH. In addition, *pta* of EcN-ato was inactivated to reduce the production of acetate. The construction resulted in a bacterial vector, designated EcN-BUT. The culturing of EcN-BUT was carried out in a similar way. Finally, EcN-BUT produced mainly biobutyrate reaching 20 mM and acetate was not detected.

### In vitro cytotoxicity of biobutyrate

**Figure 2:**
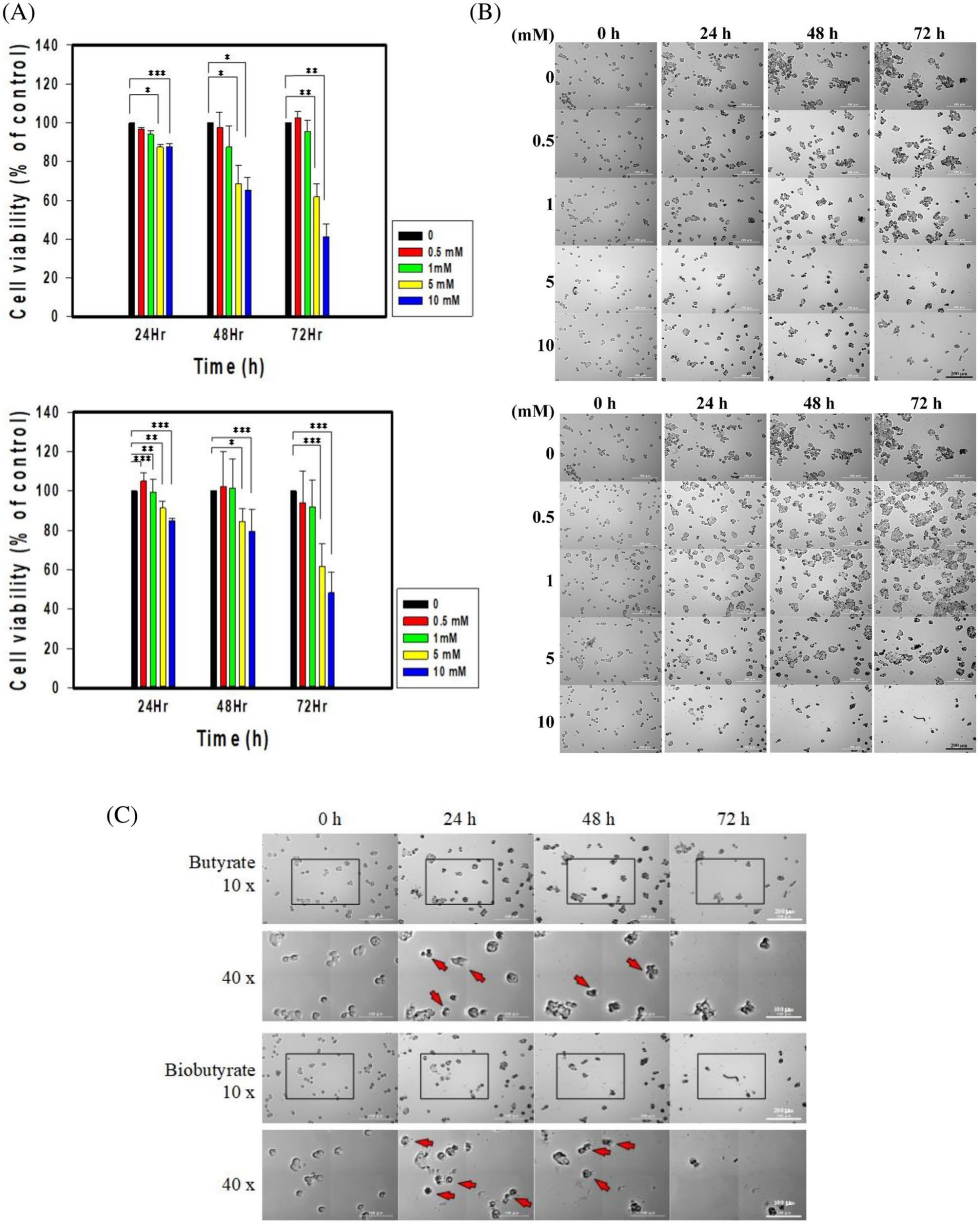
Cytotoxic effect of butyrate and biobutyrate on cancer cells. HT29 cancer cells were administrated with various concentrations of either butyrate or biobutyrate from EcN-BUT. (**A**) Cell viability by the MTT assay. The viability of HT29 cells was determined after the treatment of butyrate (top) or biobutyrate (bottom). The data was expressed as the mean ± SD from three independent experiments and analyzed by the GraphPad Prism 7 software (*p < 0.05, **p < 0.01 and ***p < 0.001 *vs*. control based on one-way ANOVA) and graphs were prepared using SigmaPlot 10.0 (Systat Software, San Jose, CA). (**B**) Cell morphology analysis by Cytation 5. Cell morphology was observed after the treatment of butyrate (top) or biobutyrate (bottom) at the indicated time. (**C**) Magnified images of cell morphology. The 10-(× 10) and 40-fold (× 40) magnified images of cell morphology were taken from (B). Apoptotic bodies were shown by arrows.

The cytotoxic effect of biobutyrate on the cancer cell was investigated with the MTT assay. Meanwhile, butyrate (Lab. chemicals) was used for comparison. HT29 cells were treated with various concentrations of biobutyrate or butyrate. As shown in Fig. 2A, the cell viability decreased in a dose- and time-dependent manner. The viability of HT29 cell that received 5 mM or 10 mM biobutyrate for 72 h dropped to 60% and 40%, respectively. Meanwhile, the treatment of butyrate at 5 mM or 10 mM caused the comparable percentage of reduction in cell viability. It indicates the bioactivity of biobutyrate. In general, the administration of 5 mM biobutyrate is sufficient to induce effective cytotoxicity against the cancer cell. The result is consistent with the previous study which reported the inhibition of cell proliferation by butyrate at a dose higher than 5 mM^45^.

The change in cellular morphology was monitored by Cytation 5. As indicated in Fig. 2B and C, cancer cells in the control group aggregated and grew over time. The cell growth was barely affected by the treatment of biobutyrate at a dose lower than 1 mM. The administration with a high dose readily caused the characteristic morphological features of apoptotic cells. Apoptotic bodies were visualized after the administration of 5 mM biobutyrate for 72 h. Cell blebs evidently appeared for HT29 cells that received 10 mM biobutyrate for 48 h. A similar pattern of the cell morphological change was observed for the butyrate treatment. As summarized in Table 1, the IC_50_ for the 24 h-, 48 h-, and 72 h-treatment of biobutyrate were estimated to be 31.55, 11.39, and 7.91 mM, respectively. Taken together, the cycotoxic effect of biobutyrate on the tumor cell was comparable to that of butyrate.

**Table 1:** IC_50_ values of biobutyrate and butyrate for HT29 cell.

	Time (h)	IC_50_ values (mM)
Butyrate	24	40.97 (4.50)
	48	19.67 (6.39)
	72	8.61 (1.72)
Biobutyrate	24	31.55 (4.08)
	48	11.39 (2.21)
	72	7.91 (2.30)

The administration time was indicated in the Table. The IC_50_ values represent the mean of at least 3 independent experiments (SD). The Excel program was applied to determine mean and SD. Following the analysis by GraphPad Prism 7, the nonlinear regression was used to calculate IC_50_. The difference between biobutyrate-based and butyrate-based IC_50_ at 24, 48, and 72 h were insignificant (*p* = 0.1916, 0.1909, and 0.7464, respectively).

### Induction of G1 phase cell cycle arrest

**Figure 3:**
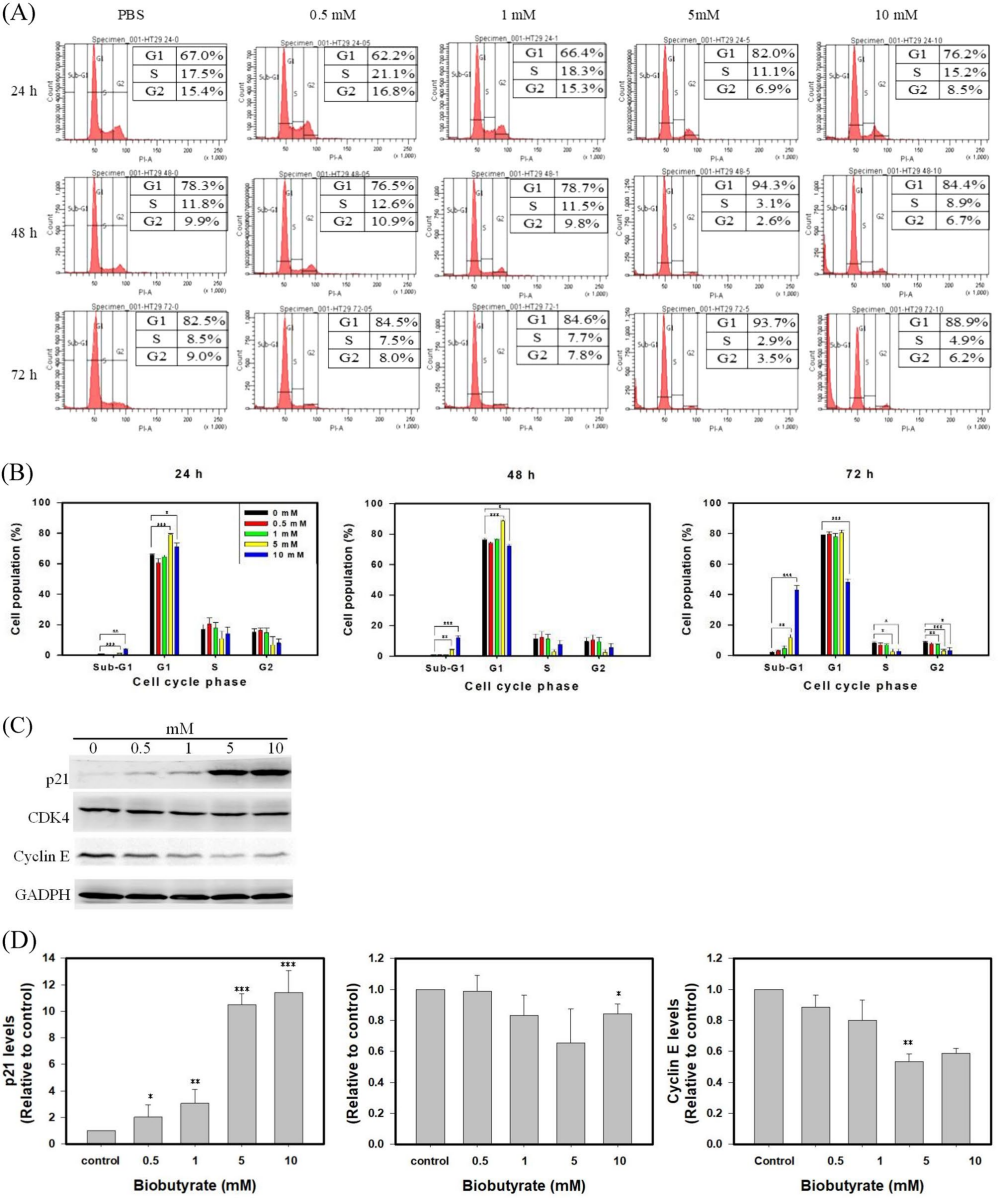
Cell cycle analysis. Cancer cells were treated with various concentrations of biobutyrate for 24, 48, and 72 h. The treatment of PBS was used as a control. (**A**) Flow cytometric analysis of biobutyrate-treated cells. After the treatment of biobutyrate, cells were analyzed by FACSCanto flow cytometer. (**B**) Quantification of cells at different stages of cell cycle. Data from three independent experiments were analyzed by the GraphPad Prism 7 software (*p < 0.05, **p < 0.01 and ***p < 0.001 *vs*. control based on one-way ANOVA) and graphs were prepared using SigmaPlot 10.0 (Systat Software, San Jose, CA). (**C**) Immunoblotting analysis of the cell cycle regulatory proteins. HT29 cells were treated with various doses of biobutyrate for 24 h. (**D**) Quantification of the cell cycle regulatory proteins. Full-length blots are presented in Supplementary Fig. 3C. Data from three independent experiments were analyzed by the GraphPad Prism 7 software (*p < 0.05, **p < 0.01 and ***p < 0.001 *vs*. control based on one-way ANOVA) and graphs were prepared using SigmaPlot 10.0 (Systat Software, San Jose, CA).

The bioactivity of biobutyrate as illustrated prompted us to investigate its effect on the cell cycle. Cancer cells were treated with various concentrations of biobutyrate and analyzed along the time course. As shown in Fig. 3A and B, cells in the G1 phase significantly accumulated when biobutyrate was administrated with a dose up to 5 mM for 24 h. The proportion of cells increased from 67.0% (control) to 82.0% (5 mM biobutyrate) and 76.2% (10 mM biobutyrate). Meanwhile, the treatment also caused a reduction in the proportion of cells in S and G2 phase of cell cycle. In the G2/M phase, cells decreased from 15.4% (control) to 6.9% (5 mM biobutyrate) and 8.5% (10 mM biobutyrate). Cells in the S phase decreased to 11.1% (5 mM biobutyrate) and 15.2% (10 mM biobutyrate) from 17.5% (control). In addition, the treatment of 10 mM biobutyrate for as early as 24 h caused a significant increase in the percentage of cells in the sub-G1 phase (apoptoic cells). It leads to an 8.28-fold (the 24-h treatment), 16.6-fold (the 48-h treatment), and 19.3-fold (the 72-h treatment) increase in the cell population in the sub-G1 phase relative to the control.

The mechanism underlying the cell cycle arrest of HT29 cells by biobutyrate was further investigated by the immunoblotting analysis (Fig. 3C,D). The treatment of biobutyrate for 24 h caused a decrease in the expression of cyclin-dependent kinase (CDK) 4 and cyclin D. In contrast, the biobutyrate-treated cells expressed a higher level of the CDK inhibitor p21 than the control level. CDK inhibitor p21 is a regulatory protein typically involved in G1 phase of cell cycle^46^. Taken together, the result suggests that biobutyrate induces cell cycle arrest at G1 phase in treated HT29 cells.

### Induction of the apoptosis pathway

**Figure 4:**
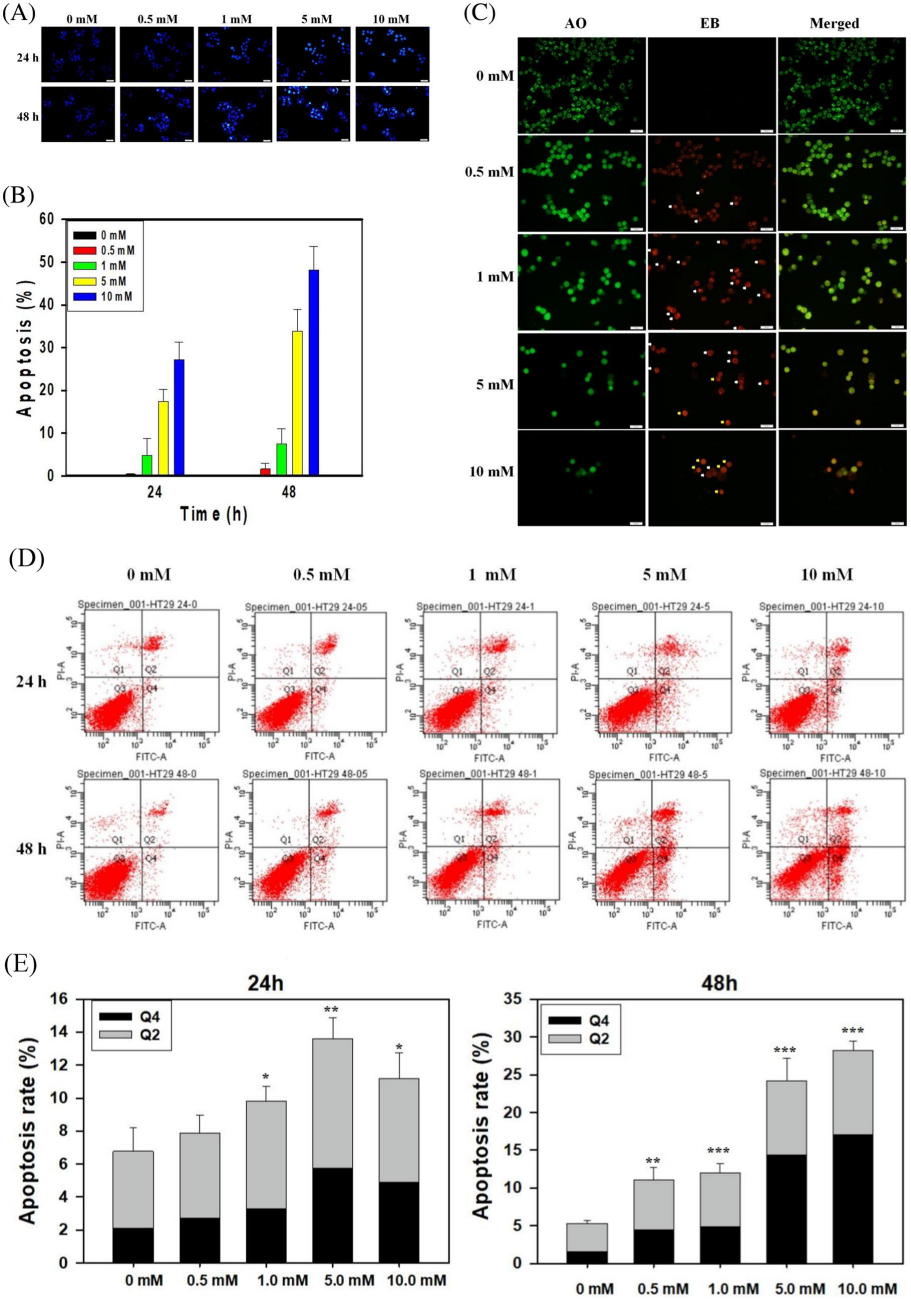
Apoptotic cell morphology. (**A**) The analysis by DAPI staining. Cancer cells were treated with various doses of biobutyrate for 24 and 48 h. Parts of apoptotic cells were marked by white and red arrows (scale bar = 50 μm). (**B**) The quantitative analysis of DAPI staining. The percentage of apoptotic cells by DAPI staining was determined after various doses of biobutyrate were administrated for indicated time and graphs were prepared using SigmaPlot 10.0 (Systat Software, San Jose, CA). (**C**) The analysis by AO/EB staining. Cancer cells were treated with various doses of biobutyrate for 72 h. Parts of early apoptotic and late apoptotic cells were marked by white and yellow arrows (scale bar = 50 μm), respectively. (**D**) Flow cytometry analysis of cell apoptosis. After being stained by Annexin V-FITC and PI, cancer cells were analyzed by FACSCanto flow cytometer. (E) Quantification of apoptotic cells involving Q2 (late apoptotic cells) and Q4 (early apoptotic cells). Data were taken from three independent experiments and analyzed using the GraphPad Prism 7 software (*p < 0.05, **p < 0.01 and ***p < 0.001 vs. control based on one-way ANOVA) and graphs were prepared using SigmaPlot 10.0 (Systat Software, San Jose, CA).

As revealed by the DAPI staining, biobutyrate-treated cells exhibited the characteristic morphology of apoptosis (Fig. 4A). After the treatment with higher biobutyrate for longer time, apoptotic cells were more frequently to show the pyknosis (shrinkage) and condensed chromatin (brighter nuclei). In contrast, the uniform but low intensity of fluorescence was detected for untreated cells. The quantitative analysis showed that the percentage of cells undergoing apoptosis reached around 50% after 10 mM biobutyrate was treated for 48 h (Fig. 4B).

The analysis of apoptotic cells was further conducted with the dual AO/EB staining. As examined by a fluorescence microscope, the nucleus of early-stage apoptotic cells was stained yellow-green and the fluorescence localized asymmetrically within treated cells. The signal of yellow-green fluorescence increased with the increasing dose of biobutyrate (Fig. 4C). Late-stage apoptotic cells clearly appeared upon the treatment of biobutyrate with a dose exceeding 5 mM. Their nuclei were marked with the orange-concentrated fluorescence.

The implementation of the Annexin V-FITC staining enables identification of apoptotic cells with the externalization of phospholipid phosphatidylserine residues. The quantitative assessment of apoptosis was determined by flow cytometry. The analysis revealed that biobutyrate induced early and late phase of apoptosis in treated HT29 cells (Fig. 4D,E). The distribution of untreated cells was mostly in Q3 (live cells). However, the cell distribution in Q2 (late apoptotic cells) and Q4 (early apoptotic cells) increased after biobutyrate was administrated. In general, the cell distribution in Q2 and Q4 increased in a dose- and time-dependent manner. The total apoptosis rates of biobutyrate (10 mM)-treated and control cells at 24 h were 11.2% (Q2:6.3%, Q4: 4.9%) and 6.7% (Q2:4.6%, Q4:2.1%), respectively. At 48 h, the total apoptosis rates of biobutyrate (10 mM)-treated and control cells increased to 28.24% (Q2: 11.17%, Q4: 17.07%) and 5.3% (Q2: 3.7%, Q4: 1.6%), respectively. In addition, the cell distribution in Q1 (necrotic cells) was barely affected by the treatment of biobutyrate. Overall, the result indicates that biobutyrate induces apoptosis in HT29 cells.

**Figure 5:**
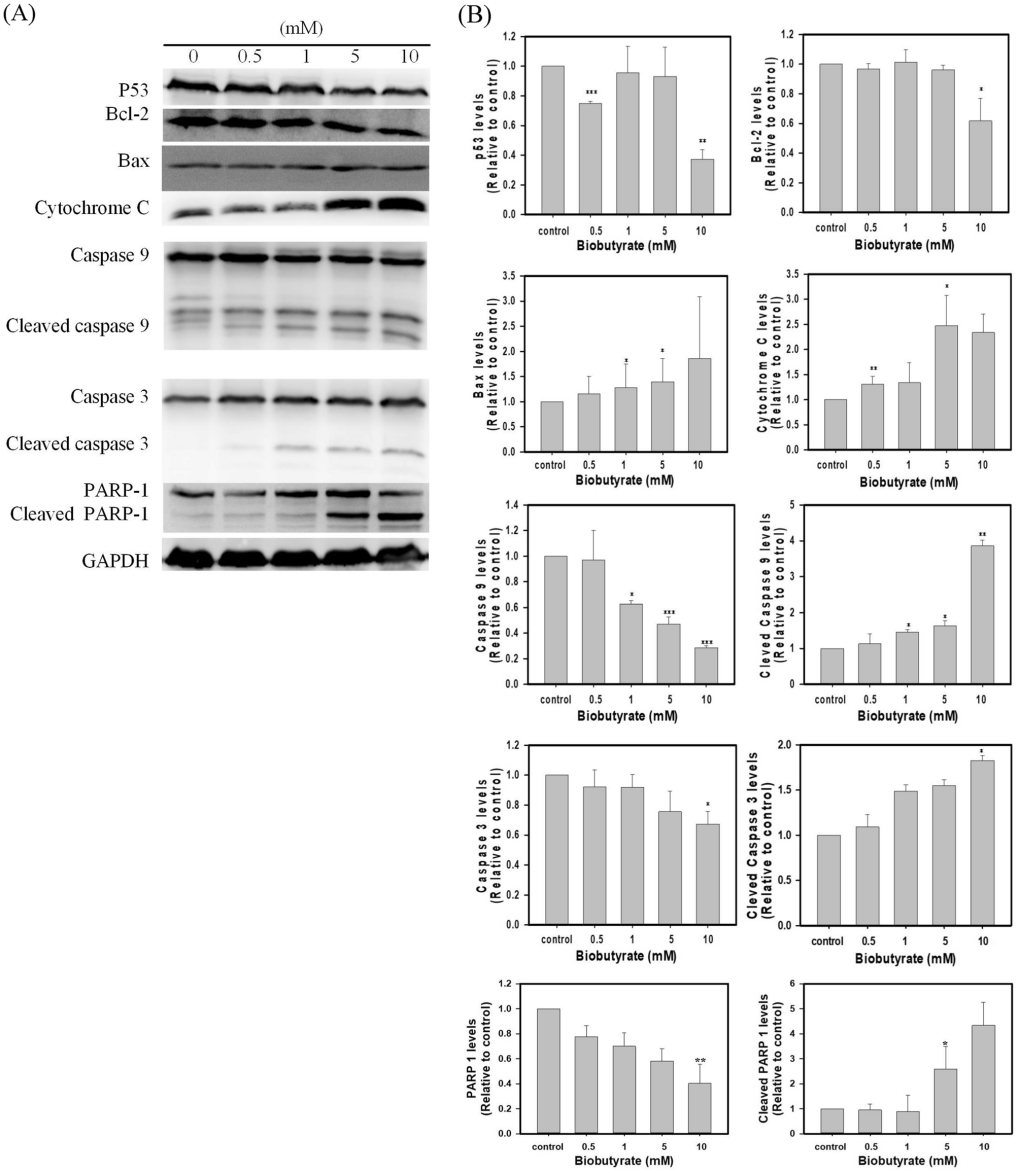
Effect of biobutyrate on the intrinsic apoptosis pathway. (**A**) The immunoblotting analysis of proteins associated with caspase-dependent apoptosis. Cancer cells were treated with various doses of biobutyrate for 48 h, and protein levels were determined by the immunoblotting analysis. The level of GADPH was used as the internal control. (**B**) Quantification of protein levels. Full-length blots are presented in Supplementary Fig. 5C. Data were taken from three independent experiments by the ImageJ Image Analysis software and analyzed using the GraphPad Prism 7 software (*p < 0.05, **p < 0.01 and ***p < 0.001 vs. vehicle control based on one-way ANOVA) and graphs were prepared using SigmaPlot 10.0 (Systat Software, San Jose, CA).

The immunoblotting analysis was implemented to investigate BCL-2 family proteins in biobutyrate-treated HT29 cells. As shown in Fig. 5A and B, the expression levels of cytochrome C, Bcl-2 associated X (BAX), and cleaved poly(ADP-ribose) polymerase 1 (PARP-1) proteins increased in a dose-dependent manner. In contrast, the treatment of 10 mM biobutyrate resulted in a down-regulation of Bcl-2. We further analyzed the effect of biobutyrate on the expression of cysteinyl-aspartate proteases (caspases). As compared to the control level, the expression levels of pro-apoptosis-related proteins including cleaved caspase-3 and cleaved caspase-9 were highly induced by the administration of biobutyrate. Moreover, the expression of the tumor suppressor p53 remained roughly unaffected but was reduced when 10 mM biobutyrate was administrated. Taken together, the result suggests that biobutyrate induces the p53-independent mitochondrial apoptosis pathway in treated HT29 cells.

### Tumor regression by biobutyrate-producing bacteria

**Figure 6:**
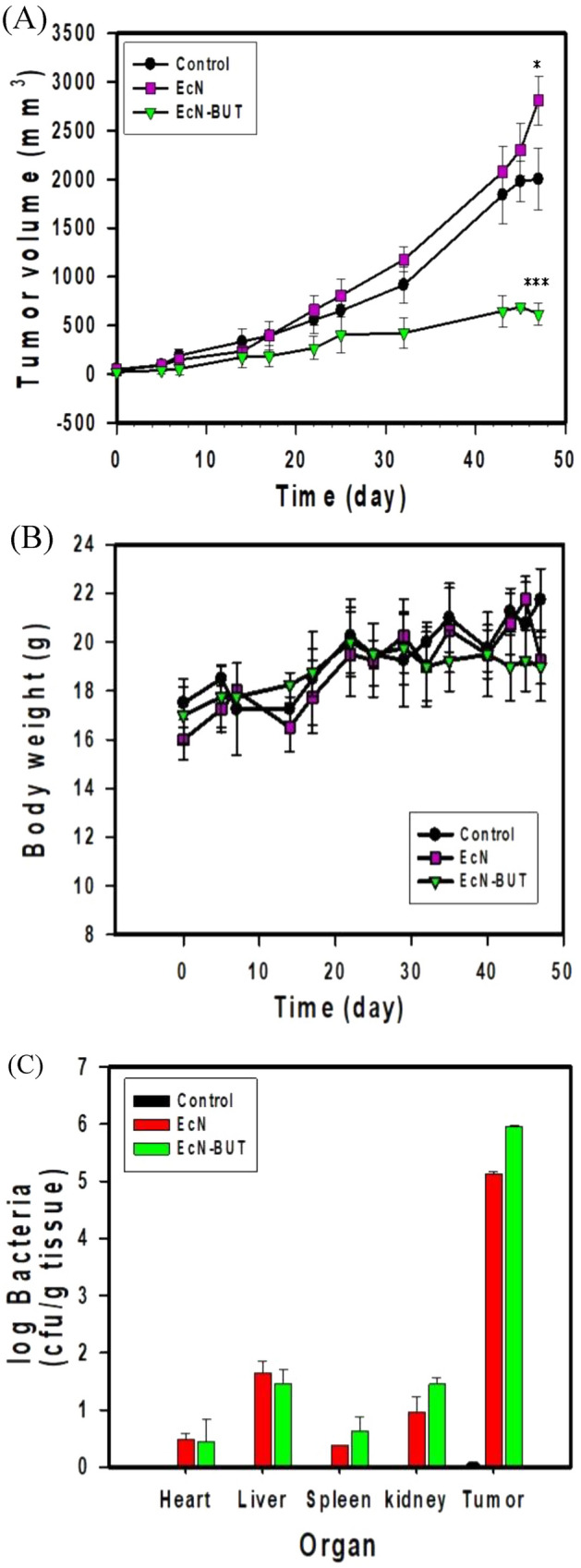
Tumor regression by EcN-BUT. (**A**) Therapeutic effect of EcN-BUT on mice (n = 5) bearing HT29 tumor cells. The tumor-bearing mice were administrated with EcN-BUT, EcN, or PBS (control). Tumor volumes (mm^3^) were estimated using external calipers, and values were expressed as means ± standard deviations. The statistical analysis was performed with Tukey's test (*p < 0.05, **p < 0.01 and ***p < 0.001) and graphs were prepared using SigmaPlot 10.0 (Systat Software, San Jose, CA). (**B**) Body weights of mice. The change in the body weight of mice was measured upon the treatment and graphs were prepared using SigmaPlot 10.0 (Systat Software, San Jose, CA). (**C**) Distribution of bacteria in vivo and graphs were prepared using SigmaPlot 10.0 (Systat Software, San Jose, CA).

The antitumor activity of the biobutyrate-producing bacterium (i.e. EcN-BUT) was investigated in tumor-bearing mice. The mice were xenografted with HT29 cells and randomly divided into three groups (n = 5). As shown in Fig. 6A, tumors in the control group continuously grew. Bacterial delivery of biobutyrate was performed by injection of EcN-BUT. This administration efficiently reduced the tumor volume by 70%. Meanwhile, the treatment of EcN (without production of biobutyrate) had no cytotoxic effect on the tumor. The result suggests the success of BCT for in situ delivery of biobutyrate. Finally, body weights of mice remained unaffected for any treatments (Fig. 6B).

The distribution of either EcN or EcN-BUT in tumor-bearing mice was determined by the bacterial CFU count. There were around 10^5^ ~ 10^6^ CFU indentified in the tumor and less than 10^2^ CFU in the organ involving heart, liver, spleen, lung, and kidney on the basis of per gram tissue (Fig. 6C). It indicates that EcN and EcN-BUT display the tumor-selective accumulation.

### Histological morphology of tumor tissues

**Figure 7:**
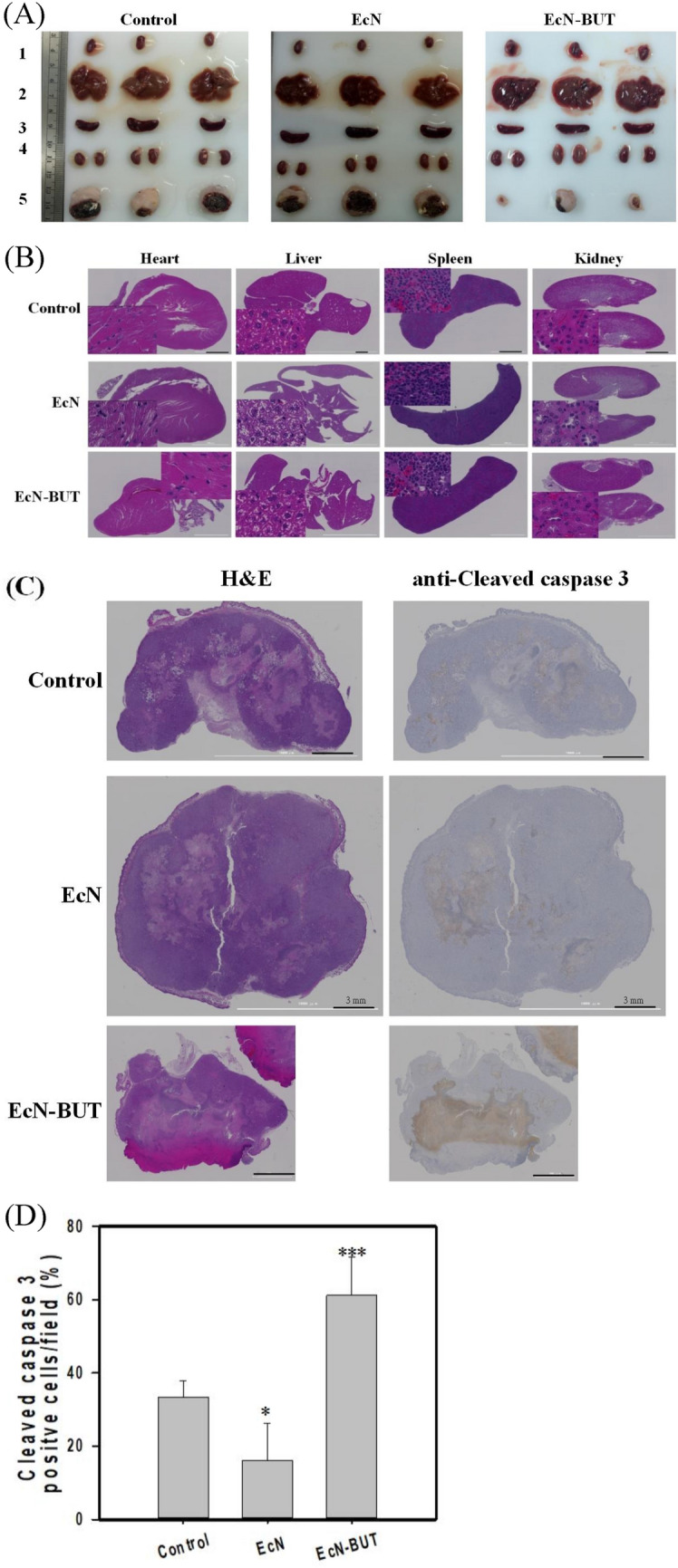
Analysis of tumor tissues and organs. (**A**) Organ and tumor sections. Photographs showed the appearance of organs and tumors size for each tested group. (**B**) H&E staining of organs. H&E staining was applied to analyze nuclear chromatin (purple-blue) and cytoplasm (red). (**C**) H&E and IHC analysis of cleaved caspase-3. The tumor-bearing mice were administrated with EcN-BUT, EcN, or PBS (control). The IHC analysis was performed by applying the anti-cleaved caspase-3 antibody to tumor tissues after the treatment. (**D**) Quantification of cleaved caspase-3. Data were taken from three independent experiments by the ImageJ Image Analysis software and analyzed using the GraphPad Prism 7 software (*p < 0.05, **p < 0.01 and ***p < 0.001 vs. control based on two-tailed t test) and graphs were prepared using SigmaPlot 10.0 (Systat Software, San Jose, CA).

The organs of each mice group were removed and shown to assume normal morphology (Fig. 7A). As illustrated by the histopathological analysis, the morphology of organs was not affected by any treatments (Fig. 7B). In particular, it had normal hepatic lobules and portal areas with intact perilobular and intralobular systems in liver sections. The result indicates that liver, spleen, heart, and kidney are not subjected to damage.

Tumor tissues were analyzed by the H&E staining. The treated-cells were characterized with more mitotic figures and angiogenesis while they exhibited disorder and different sizes and heterogeneously large nuclear regions (Fig. 7C). The nucleus of HT29 tumor in the control group displayed the blue-purple color. The color of tumor nucleus became lavender or pink in the mice group that received the treatment of EcN-BUT. The change in color indicates that the administrated agents caused the shrinkage of, the decreased density of, or the disappearance of the nucleus. The IHC analysis of the cleaved caspase-3 antibody revealed the staining intensity of each group (Fig. 7C). The apoptotic activity of cleaved caspase-3 was defined as the proportion of apoptotic cells in the tumor sections. As shown in Fig. 7D, the immunostaining intensity of EcN-BUT-treated tumor tissues was higher than that of the control. The result suggests that the activation of caspase-3 in tumors induces cell apoptosis.

## Discussion

This study proposed a novel approach of BCT for in situ delivery of a payload, biobutyrate. The core element of this technology platform is based on a bacterial vector that serves for the synthesis of and delivery of biobutyrate. EcN was chosen for development because of its probiotic nature and high efficiency of tumor-specific colonization and replication^34^. To produce biobutyrate, EcN-ato strain was equipped with the synthetic pathway consisting of endogenous *atoDA* and the heterologous gene cluster involving *phaA*, *hbd*, *crt*, and *ter*. The function of foreign genes reduces acetyl-CoA to butyryl-CoA while acetoacetyl-CoA transferase (encoded by *atoDA*) converts butyryl-CoA to biobutyrate (Fig. 1). In addition, *poxB* was knocked out by integration of *ter*. As a result, EcN-ato produced biobutyrate along with acetate. Figure 1 reveals that the *pta*- and *phaA*-mediated reaction steps compete for the acetyl-CoA pool. The result suggests that the former reaction is more active than the latter. To conserve the acetyl-CoA precursor, *pta* was inactivated in the bacterial vector. It is recognized that the redox-balanced state favors the fermentative (i.e., hypoxic) production of biobutyrate in bacteria^47^. Therefore, the approach to increase NADH was adopted by deletion of *adhE* and enhancement of *aceEF* expression. The enhanced level of *aceEF* additionally helps to direct carbon flux to acetyl-CoA. This in turn transforms the reductive pathway leading to butyryl-CoA from acetyl-CoA into an electron sink, which drives carbon flux into biobutyrate. Consequently, the resulting strain (i.e., EcN-BUT) produces primarily biobutyrate under the hypoxic condition. The limitation of *E. coli* developed for biobutyrate-based BCT is that it undergoes the mixed-acid fermentation and preferentially produces acetate^48^. Certain tumors are found to express a high level of ACSS2 which provides acetyl-CoA from acetate for the growth need^49^. To address this issue, the engineered pathway of biobutyrate utilizes acetate which is provided from the biosynthetic pathway of amino acids (Fig. 1). It therefore circumvents the surplus production of acetate after the inactivation of *pta* and *poxB*. This proposed strategy renders EcN-BUT that produces biobutyrate effective for cancer treatment (see below).

The inhibitory effect of butyrate on the development of colorectal cancer (CRC) has been extensively studied in vitro^26,50^. The dissimilation of butyrate proceeds via the β-oxidation pathway in normal cells. In contrast, cancer cells are incapable of metabolizing butyrate due to the Warburg effect^51,52^. Following entry, butyrate accumulates in the nucleus of cancer cells and acts as an inhibitor of histone deacetylase (HDAC). As a result, butyrate increases histone acetylation that regulates an array of proteins involved in cell proliferation and apoptosis. Like butyrate, biobutyrate from EcN-BUT elicits a cytotoxic effect on the human CRC cell HT29 (Fig. 2A). In line with previous reports based on butyrate^53,54^, this study showed that the administration of biobutyrate caused cell cycle arrest at the G1 phase (Fig. 3B). The regulation of cell cycle involves CDKs. In the early G1 phase, the activation of CDK4 and CDK6 by cyclin D enables phosphorylation of the retinoblastoma protein which in turn activates the transcription factor E2F to induce the necessary proteins for cell cycle^55^. By treatment of biobutyrate, HT29 cell displayed an increased level of p21 (Fig. 3D). The function of p21 inhibits the activity of cyclin D-CDK4 and cyclin D-CDK6 complexes, which renders the retinoblastoma protein non-phosphorylated and dysfunctional as well^56^. In addition, the expression level of the p53 tumor suppressor protein remained unaffected (Fig. 5B). Note that HT29 cell carries p53 mutation^57^. Taken together, the result suggests that the biobutyrate-mediated induction of p21 expression proceeds through the p53-independent route. A recent study has reported the interaction of Spl with p21^58^. Sp1 is a transcription factor that regulates genes involved in cell cycle, apoptosis, and lipogenesis, and it is deacetylated by HDAC1 and HDAC2. It is likely that biobutyrate inhibits HDAC1 and HDAC2, which in turn increases acetylation of Sp1. Acetylated Sp1 lessens its binding to the p21 promoter, consequently leading to the up-regulation of p21.

**Figure 8:**
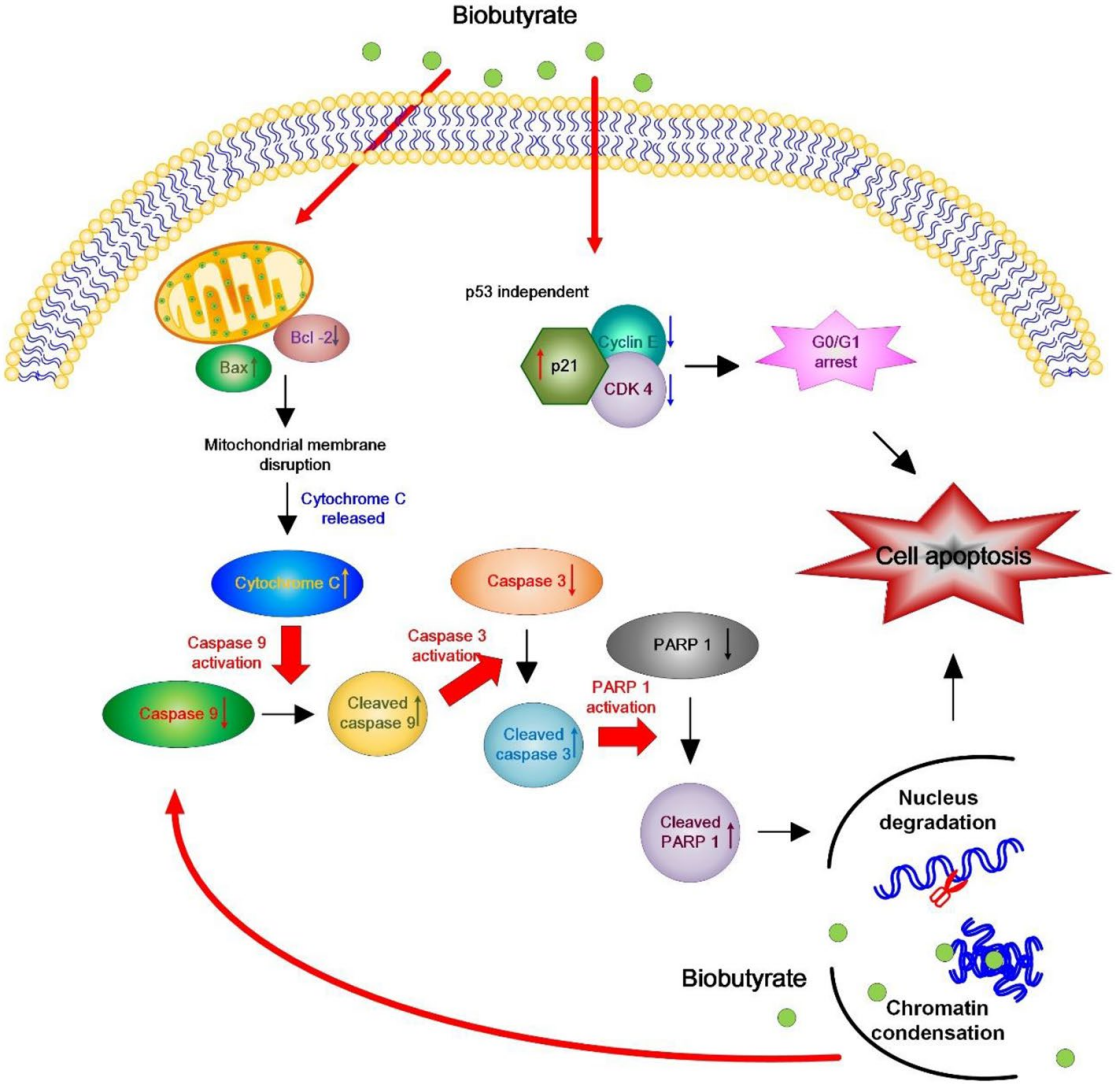
Schematic diagram showing the effect of biobutyrate on the mitochondrial apoptosis pathway and the cell cycle regulation. Biobutyrate causes the cell cycle arrest and induces cell apoptosis in HT29 cell.

The intrinsic pathway of apoptosis (i.e., the mitochondrial pathway) is typically characterized by an increase in the Bax/Bcl-2 ratio, the release of cytochrome C, and the activation of caspase-3^59^. In general, stress stimuli that perturb the normal function and regulation of mitochondria triggers mitochondrial outer membrane (MOM) permeabilization^60^. Anti-apoptotic proteins (e.g., Bcl-2) prevent the occurrence of MOM permeabilization by sequestration of pro-apoptotic proteins (e.g., Bax). The stress signals sensitize BH3-only activators to induce Bax and to suppress Bcl-2. Available Bax then undergoes conformational oligomerization and integrates into MOM concomitant with the formation of pores through which cytochrome C is released^61^. In cytoplasm, apoptosome appears as a result of the interaction of cytochrome C with apoptotic protease-activating factor 1 (APAF-1). Following the activation by apoptosome, caspase-9 processes caspase-3 which cleaves structural proteins, cell cycle proteins, and DNase proteins to cause cell death^62^. In this study, the administration of biobutyrate induced apoptosis of HT29 cell. The analyses illustrated that the apoptotic event arose in association with the down-regulation of Bcl-2 and the up-regulation of Bax, cytochrome C, cleaved caspase-9, and cleaved caspase-3 (Fig. 5B). There was no significant variation in the expression level of p53. Interestingly, an increased level of cleaved PARP-1 was detected. PARP-1 has a broad range of physiological and pathological functions involved in the balance between cell survival and cell death^63^. It is a nuclear protein that routinely facilitates the repair of DNA damage. By cleavage of suicide proteases (e.g., caspases), PARP-1 is split into various signature fragments that participate in the certain process of pathological cell death. The previous study showed that active caspase-3 cleaves PARP-1 to produce an N-terminal DNA-binding domain which acts as a transdominant inhibitor of active PARP-1. This in turn promotes cell apoptosis by attenuation of DNA repair^28^. Taken together, it is likely that biobutyrate triggers the mitochondrial pathway independent of p53. As summarized in Fig. 8, biobutyrate increases the expression of p21 which causes the cell cycle arrest by inhibition of CDKs and cyclin E involved in the early and the mid G1 phase, respectively. In addition, biobutyrate inhibits the expression of Bcl-2 while it increases the expression of Bax. This result leads to the liberation of cytochrome C. The formation of apoptosome then activates caspase-9 that processes caspase-3 to split PARP-1, which consequently induces cell apoptosis.

In tumor-bearing mice, the implementation of EcN-BUT effectively reduced the volume of HT29 tumor by 70% (Fig. 6A). It suggests that a bioactive dose of biobutyrate reaches the cancerous site. The identification of cleaved caspase-3 by IHC suggests that delivered biobutyrate induces apoptosis of tumor cells via the intrinsic pathway (Fig. 7D). In addition, EcN and its derivative EcN-BUT exhibited the tumor-specific colonization at the tumor-to-organ ratio of 10^4^:1. The application of EcN for BCT based on the protein payload has been demonstrated in various cancer cell lines, including 4T1 breast cancer cell^64^, B16 melanoma cell^64^, and SMMC-7721 hepatoma cell^65^. The mechanism through which EcN targets tumors remains unclear. Nevertheless, EcN has an extracellular structure composed of K5 capsule^66^ and F1C fimbriae^67^ which facilitate its intestinal colonization. It is apparent that a high density of colonized EcN-BUT competes with tumors for nutrients and oxygen. Following the decrease of available oxygen, a hypoxic condition arises in favor of the fermentative synthesis of biobutyrate in EcN-BUT. The production of biobutyrate continues with the increasing formation of the apoptotic regions, followed by the movement of EcN-BUT toward the tumor peripheral. Consequently, the volume of HT29 tumor notably regressed.

## Conclusions

The beneficial effect of butyrate has been illustrated to reduce inflammation and carcinogenesis. This issue was addressed by development of BCT in this study. By metabolic engineering, probiotic EcN was finely reprogrammed to mainly produce biobutyrate. This strategy offer the following advantages over others: it allows (1) in vivo and continuous production of the therapeutic agent (i.e., biobutyrate) without prior purification and complicated formulation for use, (2) in situ and targeted delivery of biobutyrate to reduce side effects and improve bioavailability. As a result, the implementation of BCT caused the significant regression of the tumor volume. In particular, this approach enables simultaneous production of more than one payload. The administration of butyrate in combination with other agents has been exploited to increase the intervention efficacy in cancer cells^68^. Therefore, it would be intriguing to learn the potential of BCT based on the biobutyrate-producing EcN-BUT that additionally produces, for instance, a cytotoxic protein payload. In particular, the anti-cancer effect of butyrate is not limited to CRC cells and is manifest in breast cancer cell^69^, leukemia cell^70^, ovarian cancer cell^71^, and prostate cancer cell^72^. Taken together, the technological advance in synthetic biology facilities the elaborate design of a robust biobutyrate-based BCT for medical intervention of various tumors. The present study paves the way to realizing this goal.

## Supplementary Information

Supplementary Information.
