# Supplementary material for: In situ delivery of biobutyrate by probiotic Escherichia coli for cancer therapy
Source: Sci Rep. 2021 Sep 13;11:18172. doi: 10.1038/s41598-021-97457-3 (PMC8438071; doi:10.1038/s41598-021-97457-3)

In situ delivery of biobutyrate by probiotic Escherichia coli for cancer therapy

Chung-Jen Chiang,[1,*], Yan-Hong Hong [2]

[1]Department of Medical Laboratory Science and Biotechnology, China Medical University, No. 91, Hsueh-Shih Road, Taichung, Taiwan 40402

[2]Department of Chemical Engineering, Feng Chia University, Taichung, Taiwan 40724

*Address correspondence to:

Dr. Chung-Jen Chiang

E-mail: cjchiang@mail.cmu.edu.tw

TEL: 886-4-22003366 ext. 7227

Fax: 886-4-22057414

Supplementary Dataset File

Unedited blots and replicates of cell cycle-related biomarkers in the figure 3c. Blots of p21, CDK4, Cyclin E, and GAPDH were cut prior to hybridization with antibodies.

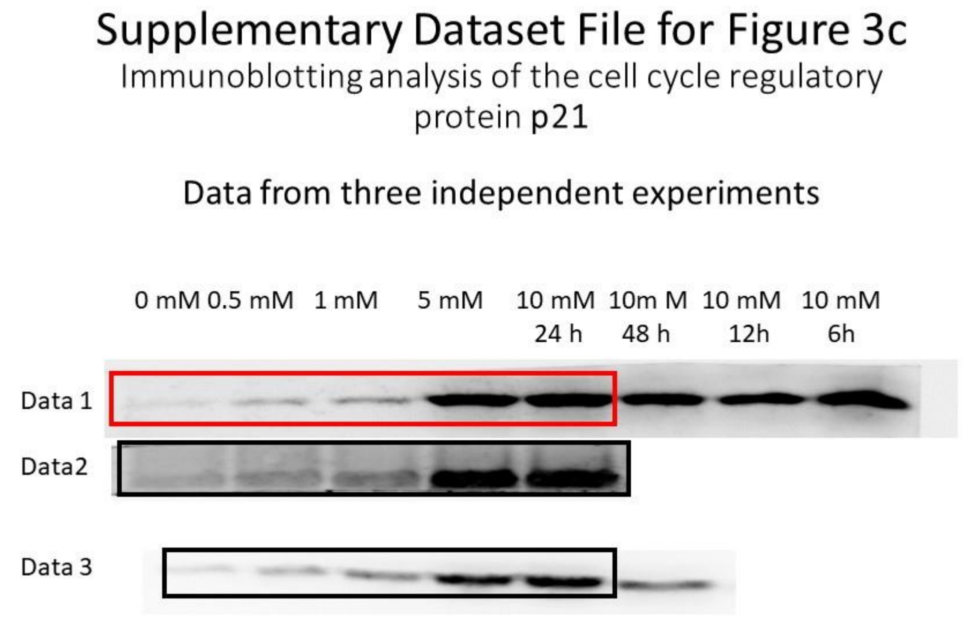

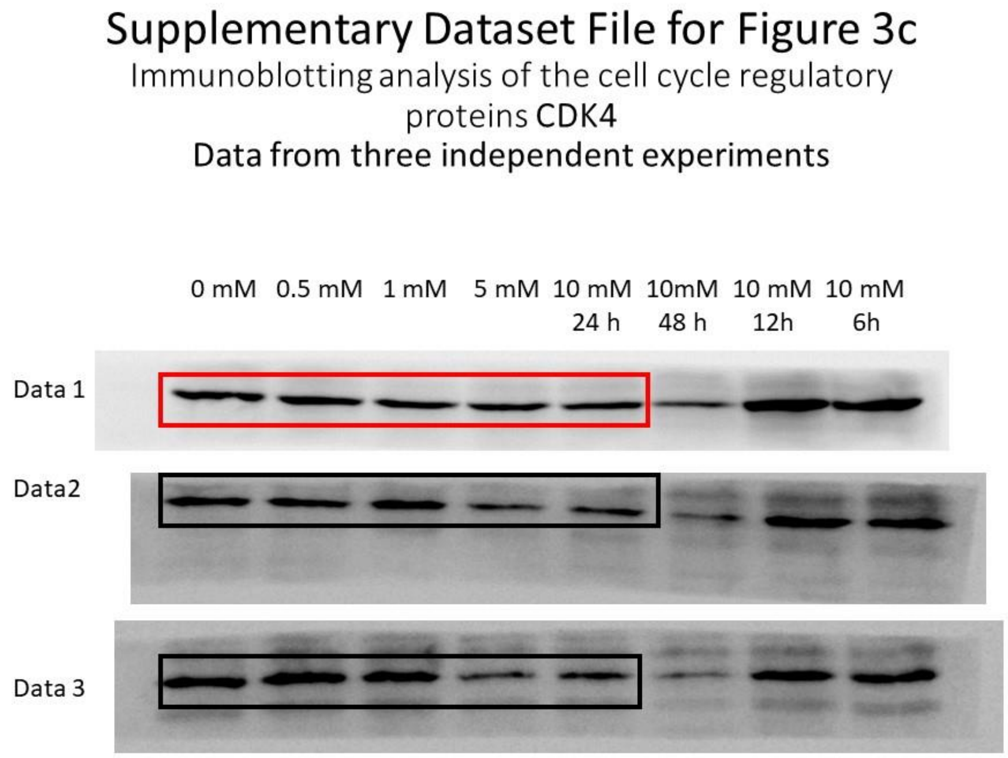

Supplementary Dataset File for Figure 3c

Immunoblotting analysis of the cell cycle regulatory protein **Cyclin E**

Data from three independent experiments

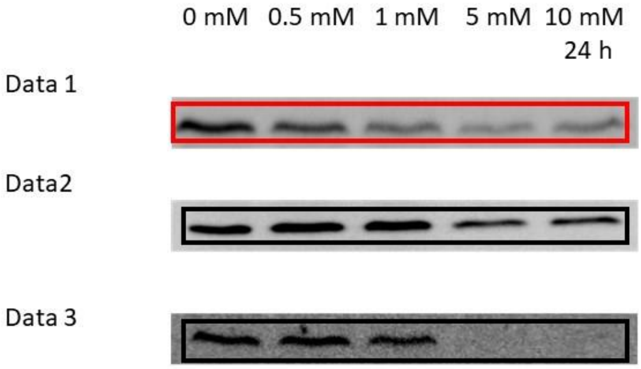

Supplementary Dataset File for Figure 3c

Immunoblotting analysis of the cell cycle regulatory protein **GAPDH**

Data from three independent experiments

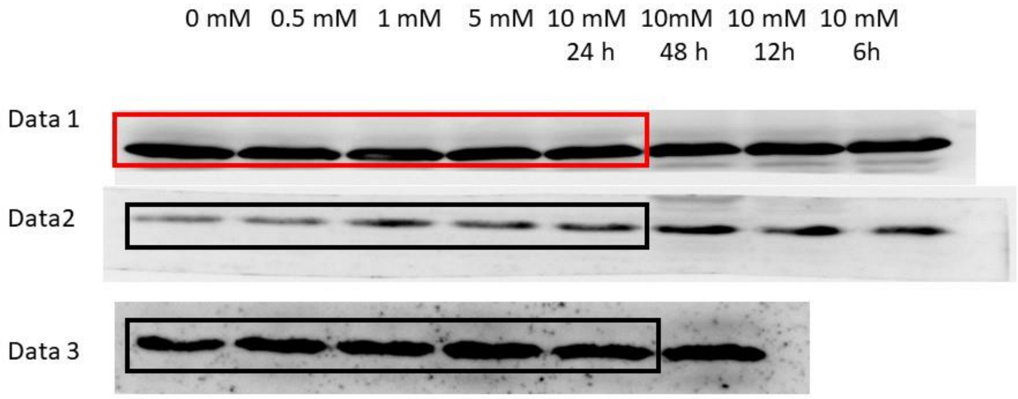

Unedited blots and replicates of apoptosis-related biomarkers in the figure 5a. Blots of p53, BCL-2, Bax, Cytochrome C, Caspase 9, Caspase 3, PARP, and GAPDH were cut prior to hybridization with antibodies.

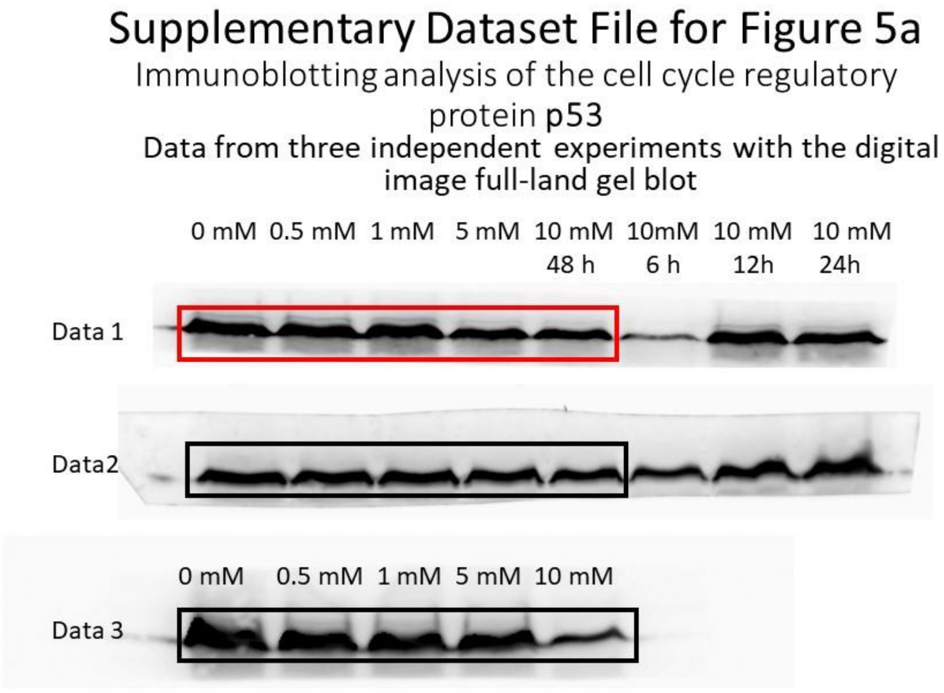

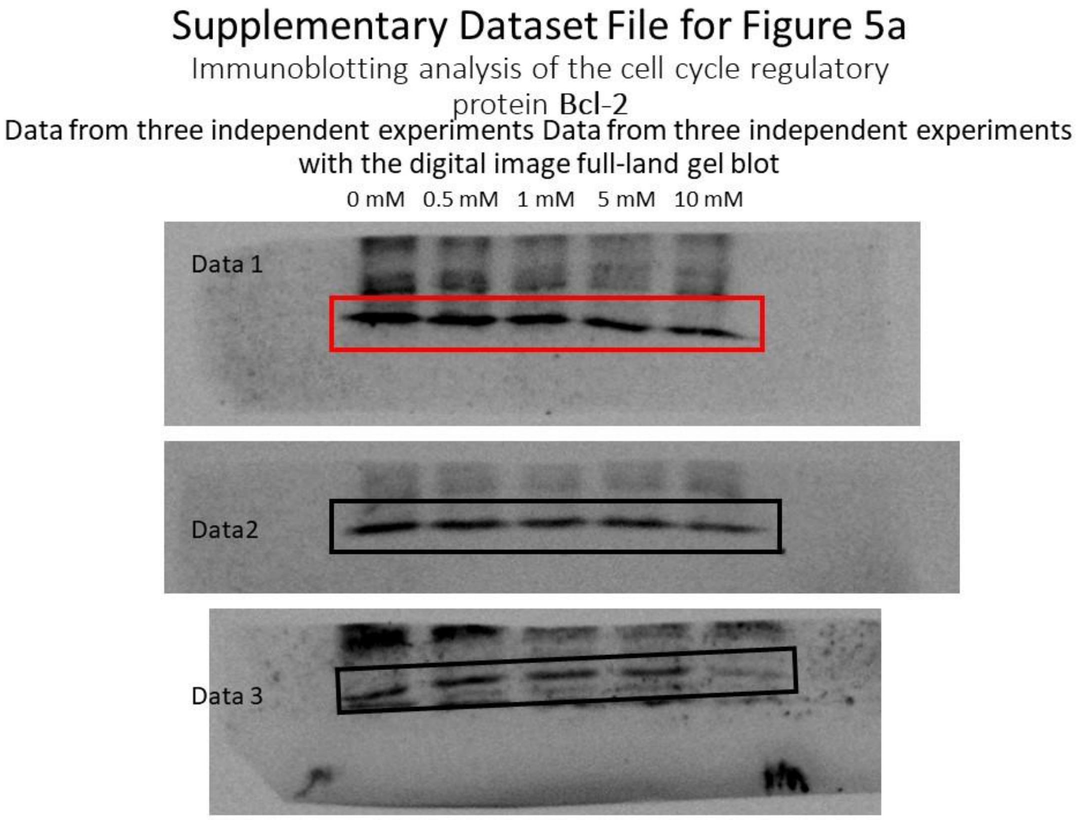

Supplementary Dataset File for Figure 5a

Immunoblotting analysis of the cell cycle regulatory protein **Bax**

Data from three independent experiments

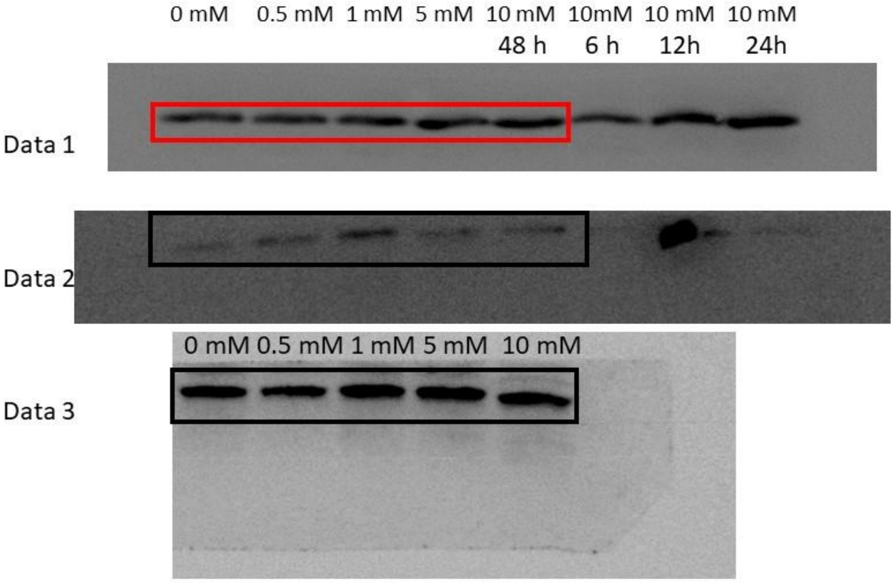

Supplementary Dataset File for Figure 5a

Immunoblotting analysis of the cell cycle regulatory protein **Cytochrome C**

Data from three independent experiments

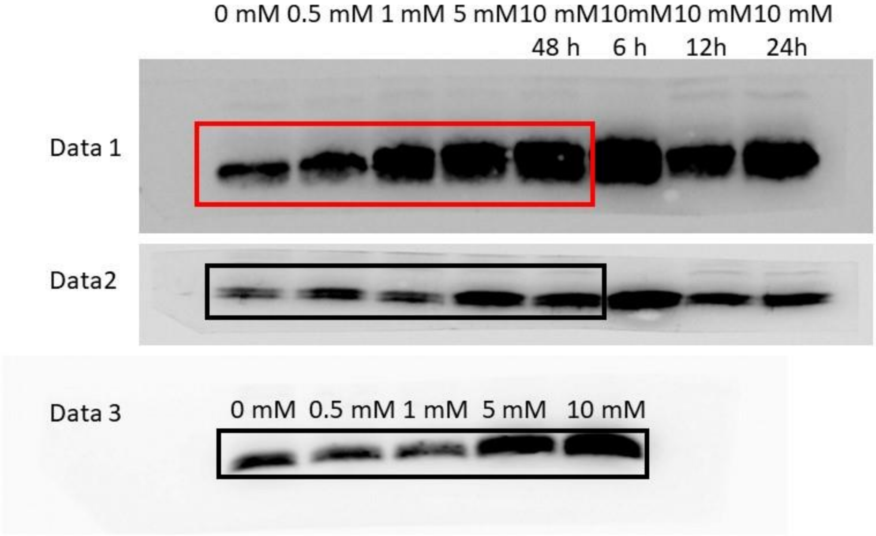

Supplementary Dataset File for Figure 5a

Immunoblotting analysis of the cell cycle regulatory protein Caspase 9 and cleaved Caspase 9

Data from three independent experiments

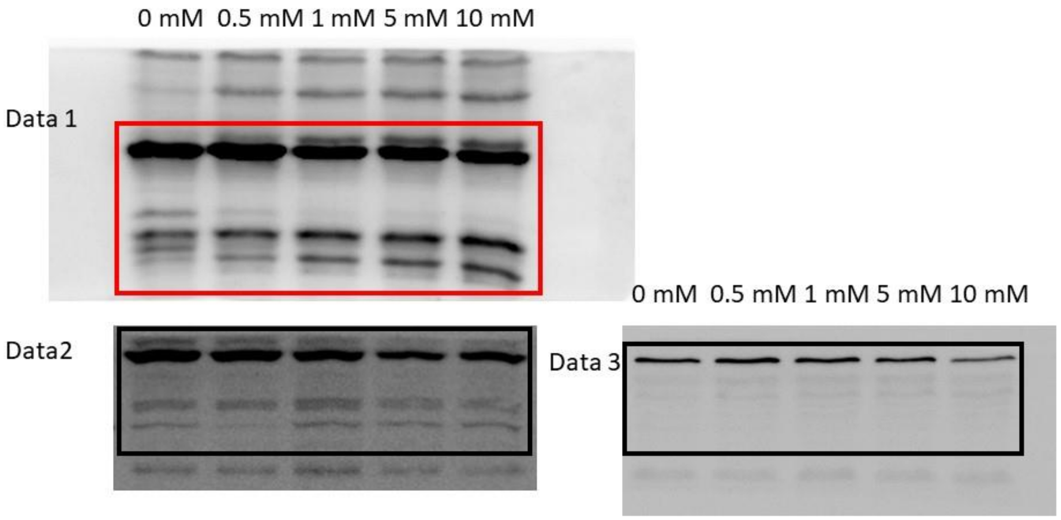

Supplementary Dataset File for Figure 5a

Immunoblotting analysis of the cell cycle regulatory protein Caspase 3 and cleaved Caspase 3

Data from three independent experiments

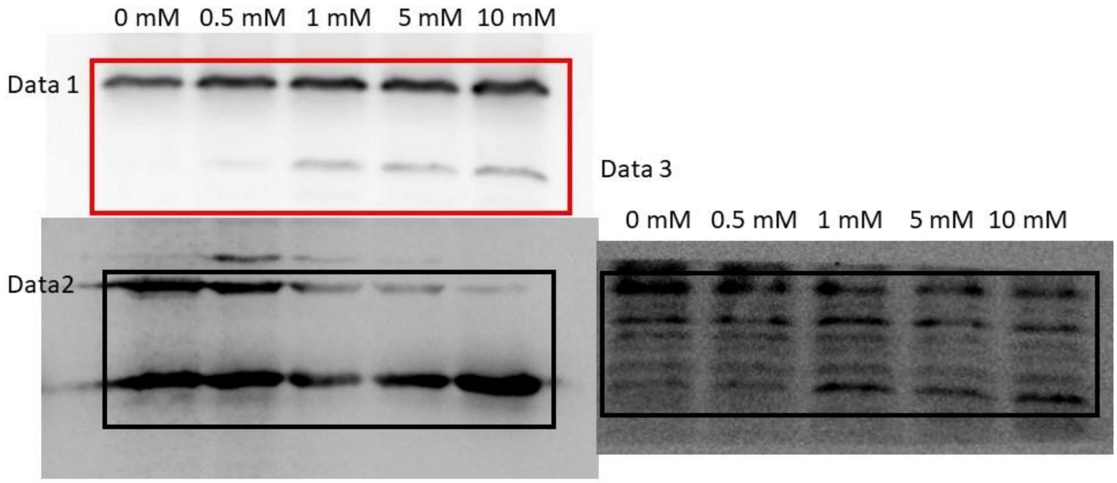

Supplementary Dataset File for Figure 5a

Immunoblotting analysis of the cell cycle regulatory protein **PARP-1 and Cleaved PARP-1**

Data from three independent experiments

0 mM 0.5 mM 1 mM 5 mM 10 mM 48 h 10mM 6 h 10 mM 12h 10 mM 24h

Data 1

Data 2

Data 3’

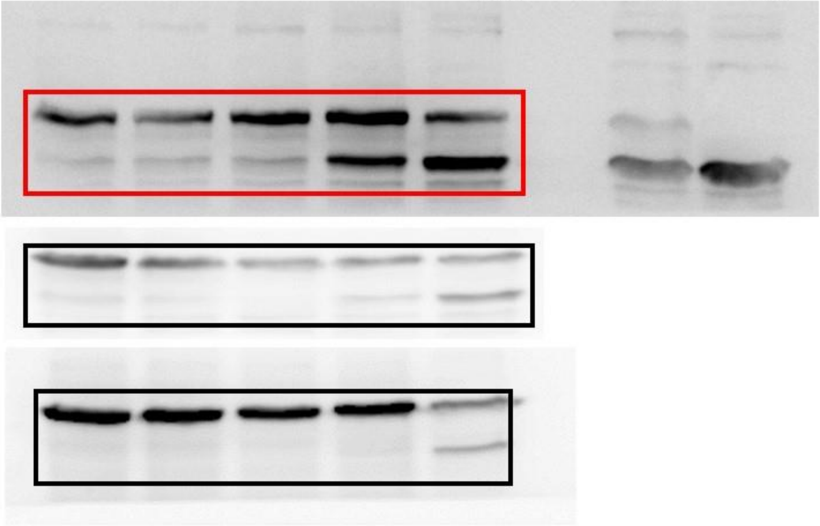

Supplementary Dataset File for Figure 5a

Immunoblotting analysis of the cell cycle regulatory protein **GAPDH**

Data from three independent experiments for P53, Bax, Cytochrome C and PARP-1 and Cleaved PARP-1

0 mM 0.5 mM 1 mM 5 mM 10 mM 48 h 10mM 6 h 10 mM 12h 10 mM 24h

Data 1

Data2

Data 3

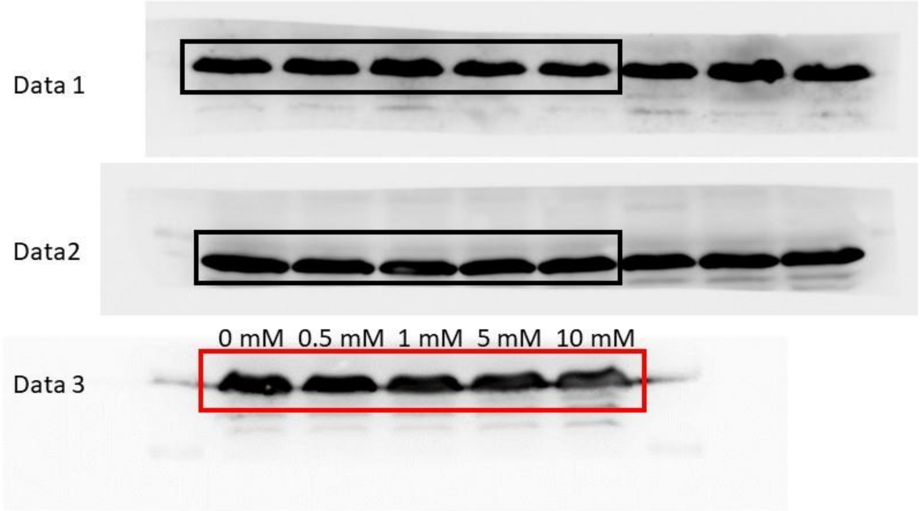

Supplementary Dataset File for Figure 5a

Immunoblotting analysis of the cell cycle regulatory protein GAPDH

Data from three independent experiments for Bcl-2, Caspase 9, cleaved Caspase 9, and Caspase 3, cleaved Caspase 3

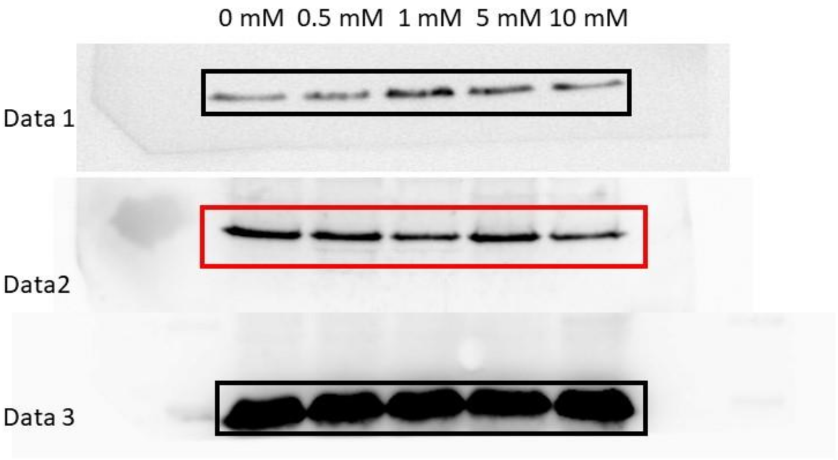

Supplement: Supplementary file 1 — Supplementary Information. [file 41598_2021_97457_MOESM1_ESM.pdf]
